# Synthesis of Some Novel Fused Pyrimido[4″,5″:5′,6′]-[1,2,4]triazino[3′,4′:3,4] [1,2,4]triazino[5,6-*b*]indoles with Expected Anticancer Activity

**DOI:** 10.3390/molecules23030693

**Published:** 2018-03-19

**Authors:** Rania S. Ali, Hosam A. Saad

**Affiliations:** 1 Chemistry Department, Faculty of Science, Taif University, Taif 21974, Saudi Arabia; h_saadzag@yahoo.com; 2 Department of Basic Science, Faculty of Industrial Education, Helwan University, Cairo 11795, Egypt; 3 Chemistry Department, Faculty of Science, Zagazig University, Zagazig 44511, Egypt

**Keywords:** triazino[5,6-*b*]indole, pyrimido[2′,1′:3,4]triazino[5,6-*b*]indole, anticancer activity, molecular docking

## Abstract

Our current goal is the synthesis of polyheterocyclic compounds starting from 3-amino-[1,2,4]triazino[5,6-*b*]indole **1** and studying their anticancer activity to determine whether increasing of the size of the molecules increases the anticancer activity or not. 1-Amino[1,2,4]triazino[3′,4′:3,4]-[1,2,4]triazino[5,6-*b*]indole-2-carbonitrile (**4**) was prepared by the diazotization of 3-amino[1,2,4]-triazino[5,6-*b*]indole **1** followed by coupling with malononitrile in basic medium then cyclization under reflux to get **4**. Also, new fused pyrimido[4″,5″:5′,6′][1,2,4]triazino-[3′,4′:3,4][1,2,4]triazino[5,6-*b*]indole derivative **6** was prepared and used to obtain polycyclic heterocyclic systems. Confirmation of the synthesized compounds’ structures was carried out using elemental analyses and spectral data (IR, ^1^H-NMR and ^13^C-NMR and mass spectra). The anticancer activity of some of the synthesized compounds was tested against HepG2, HCT-116 and MCF-7 cell lines. The anticancer screening results showed that some derivatives display good activity which was more potent than that of the reference drug used. Molecular docking was used to predict the binding between some of the synthesized compounds and the prostate cancer 2q7k hormone and breast ‎cancer 3hb5 receptors.

## 1. Introduction

**Figure 1:**
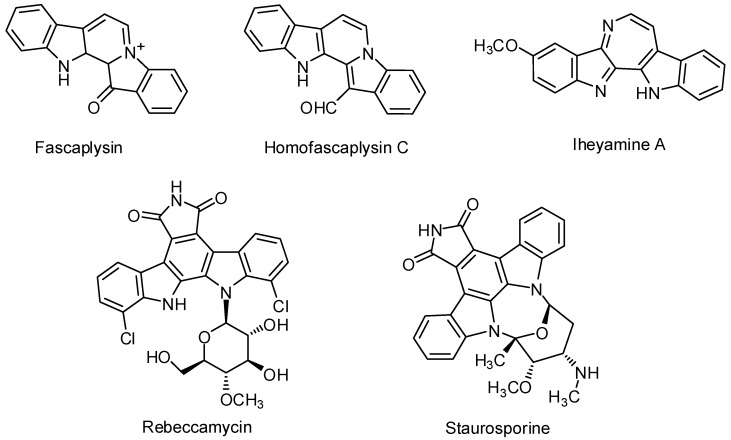
Some active poly cyclic natural compounds.

Interestingly, we are now seeing the emergence of a new generation of drugs with hybrid molecular architectures combining the biological features of two or more small molecules. The expectation is that, in the long term, such molecular conjugates could become a dominant form of targeted infectious diseases. The idea of combining two or more potentially bioactive substructures to make an integrated new molecular framework with a higher anticipated therapeutic power is conceptually simple yet highly successful in the context of infectious diseases that require effective treatment. In previous works, we succeeded in constructing many small 1,2,4-triazine molecules that had a remarkable biological activity on cancer cells [1,2]. In addition, we were able to construct many fused 1,2,4-triazine systems which showed marvelous antimicrobial activities [3,4,5,6,7,8,9,10]. Herein, we aspire to constructed huge fused triazine molecules of increased efficacy and decreased systemic toxicity which are a great challenge for medical science. One of the present goals in heterocyclic compound synthesis is the construction of macromolecules, in particular, by using of sequential reactions [11]. Such molecular explorations, yielding novel architectures, are of particular interest for the investigation of new bioactive agents with possible new modes of action, which could subsequently be elaborated in medicinal chemistry programs [12]. Approaches to the realization of these synthetic goals have often been explored in the context of complex multistep syntheses of natural product targets [13]. Among the various clinical applications, heterocyclic compounds have a considerable active role as anti-viral [14], anti-bacterial [15,16], anti-inflammatory [17], anti-fungal [18], and anti-tumor drugs [19,20,21]. Numerous of biologically active macromolecules naturally occurring (e.g., with antimicrobial activity or anticancer) include homofascaplysin [22], fascaplysin (antimicrobial pigment) [23,24,25,26], staurosporine [27], iheyamines [28], and rebeccamycin [29] are shown in Figure 1.

The amazing biological activity of the polycyclic heterocyclic compounds encouraged us to continue our previous work on the synthesis of fused triazine[7,9,10] and their applications, by designing a polycyclic heterocyclic compounds containing five and/or six rings fused with each other in the hope to get a superior biological activity.

## 2. Results and Discussion

**Scheme 1:**
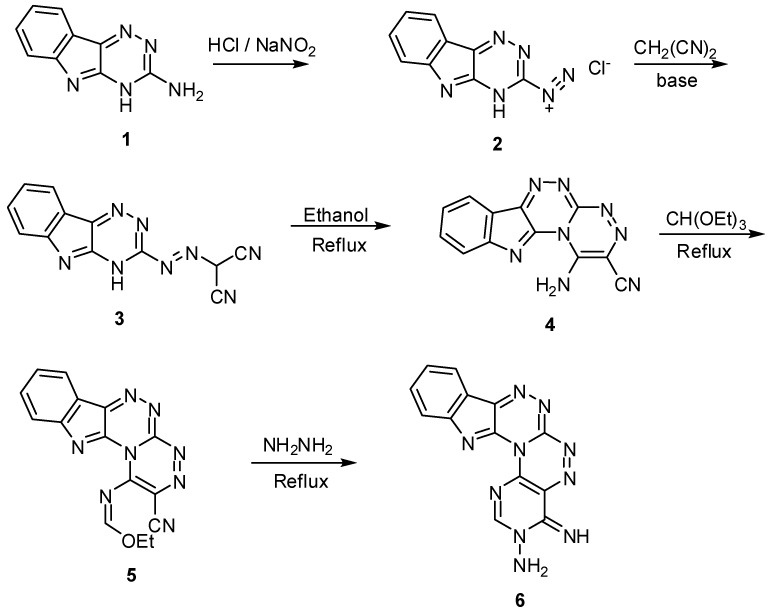
Synthesis of 4-iminopyrimido[4″,5″:5′,6′][1,2,4]triazino[3′,4′:3,4][1,2,4]triazino-[5,6-*b*]indol-3(4*H*)-amine 6.

The previously prepared 3-amino-4*H*-[1,2,4]triazino[5,6-*b*]indole **1** [30] was used in this work as a starting unit on which the polycyclic compound will built. Compound **1** was subjected to diazotization at 4 °C in conc. HCl with aqueous solution of NaNO_2_ (Scheme 1). To the stirred solution an aqueous mixture of malononitrile and sodium acetate was added to obtain [4H-[1,2,4]triazino[5,6-*b*]indol-3-yldiazenyl]malononitrile (**3**). The structure of **3** was elucidated from its IR spectrum, which showed two cyano groups signals at 2210, 2217 cm^−1^ with disappearance of the band corresponding to the NH_2_ group of compound **1**. Cyclization of compound **3** to 1-amino[1,2,4]triazino[3′,4′:3,4]-[1,2,4]triazino[5,6-*b*]indole-2-carbonitrile (**4**) was achieved by refluxing compound **3** in ethanol. The structure elucidation of compound **4** was supported from the disappearance of one cyano group peak in the IR, as only one CN group was detected at 2209 cm^−1^, in addition to the disappearance of the triazine NH peak at 3201 cm^−1^ along with appearance of a NH_2_ group in the 3351–3313 cm^−1^ range. The ^1^H-, ^13^C-NMR data supported the same results, where the disappearance of the NH signal of compound **3** at δ = 11.42 ppm was compensated by the appearance of the NH_2_ signal of compound **4** at δ = 6.90 ppm. The ^13^C-NMR data, where one of the CN signals disappeared, also advocated the same results. Refluxing of compound **4** with triethyl orthoformate in ethanol resulted in the disappearance of the NH_2_ signals in both the IR and ^1^H-NMR spectra, respectively, due to the formation of the compound ethyl (2-cyano[1,2,4]triazino[3′,4′:3,4][1,2,4]triazino[5,6-*b*]indol-1-yl)imidoformate (**5**) with appearance of ethyl group signals in the ^1^H- and ^13^C-NMR, respectively. Compound **5** was subjected to reaction with hydrazine hydrate in boiling ethanol to get the first fifth cyclic compound 3(4*H*)-amino-4-iminopyrimido[4″,5″:5′,6′][1,2,4]triazino[3′,4′:3,4][1,2,4]triazino[5,6-*b*]indole (**6**), which was considered as a second starting material for the formation of six-ring cyclic compounds (Scheme 1). 

**Scheme 2:**
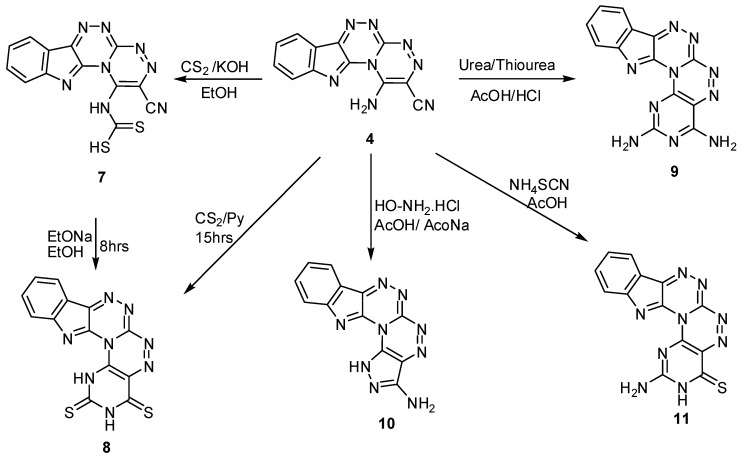
Some reactions on 1-amino[1,2,4]triazino[3′,4′:3,4][1,2,4]triazino[5,6-*b*]indole-2-carbonitrile.

Conformation of the structure of compound **6** was achieved from it ^1^H-NMR, where the signals corresponding to the ethyl group of compound **5** at δ = 1.26 and 3.51 ppm disappeared and new bands due to NH_2_ and NH at δ = 5.83 and 10.13 ppm appeared, while the ^13^C-NMR showed the disappearance of the sp^3^ carbons of the ethyl group of compound **5**. The first starting material, **4**, reacted with carbon disulphide in alcoholic KOH under reflux, to get the carbamodithioic acid derivative **7** which was cyclized to give pyrimido[4″,5″:5′,6′][1,2,4]triazino[3′,4′:3,4][1,2,4]triazino[5,6-*b*]indole-2,4(1*H*,3*H*)-dithione (**8**) by refluxing compound **7** in hot ethanolic sodium ethoxide for 8 h. Compound **8** was also prepared by directly refluxing compound **4** with CS_2_ in boiling pyridine. The structures of compounds **7** and **8** were confirmed by their spectral analysis, where the ^1^H-NMR of compound **7** showed the appearance of a signal at δ = 13.98 ppm due to a SH group, which disappeared in compound **8** and 2 NH signals shown at δ = 11.40 and 12.50 ppm, respectively (Scheme 2).

**Scheme 3:**
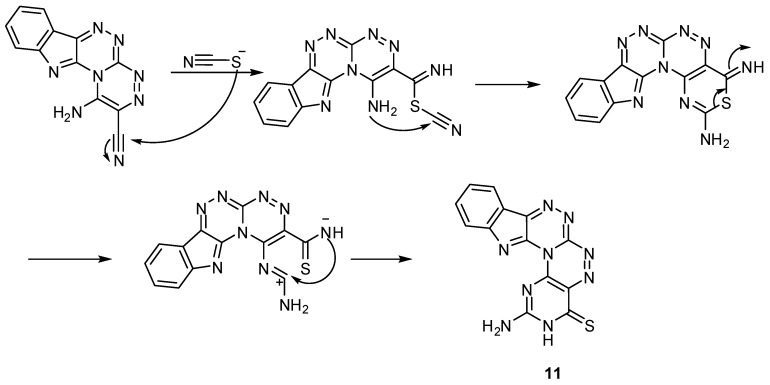
Mechanism that explains the formation of compound **11**.

Reactions of compound **4** with urea and/or thiourea in boiling glacial acetic acid in presence of a catalytic amount of HCl yielded 2,4-diaminopyrimido[4″,5″:5′,6′][1,2,4]triazino[3′,4′:3,4][1,2,4]-triazino[5,6-*b*]indole (**9**), while 3-amino-1*H*-pyrazolo[3″,4″:5′,6′][1,2,4]triazino[3′,4′:3,4]-[1,2,4]-triazino-[5,6-*b*]indole (**10**) and 2-aminopyrimido[4″,5″:5′,6′][1,2,4]triazino[3′,4′:3,4]-[1,2,4]triazino-[5,6-*b*]indole-4(3*H*)-thione (**11**) were formed by the reactions of **4** with hydroxylamine hydrochloride in glacial acetic acid containing anhydrous sodium acetate as a catalyst and ammonium thiocyanate in glacial acetic acid. The structures of compounds **9**, **10** and **11** were confirmed from their spectral data, where the IR of all compounds showed the disappearance of the cyano group peak with appearance of peaks corresponding to NH_2_, NH in the 3413–3754 cm^−1^ range, and the IR of compound **11** showed a C=S moiety at 1349 cm^−1^. The ^1^H-NMR of the three compounds show signals due to NH_2_ and in some NH signals in the δ = 5.55–6.93 and 11.12–12.33 ppm ranges, respectively (Scheme 2). The mechanism proposed for the formation of compound **11** is illustrated in Scheme 3.

**Scheme 4:**
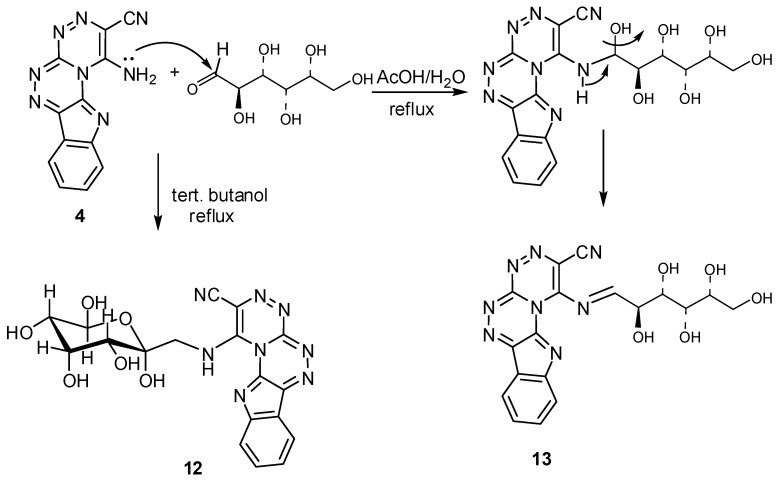
Reactions of 1-amino[1,2,4]triazino[3′,4′:3,4][1,2,4]triazino[5,6-*b*]indole-2-carbonitrile with glucose.

**Scheme 5:**
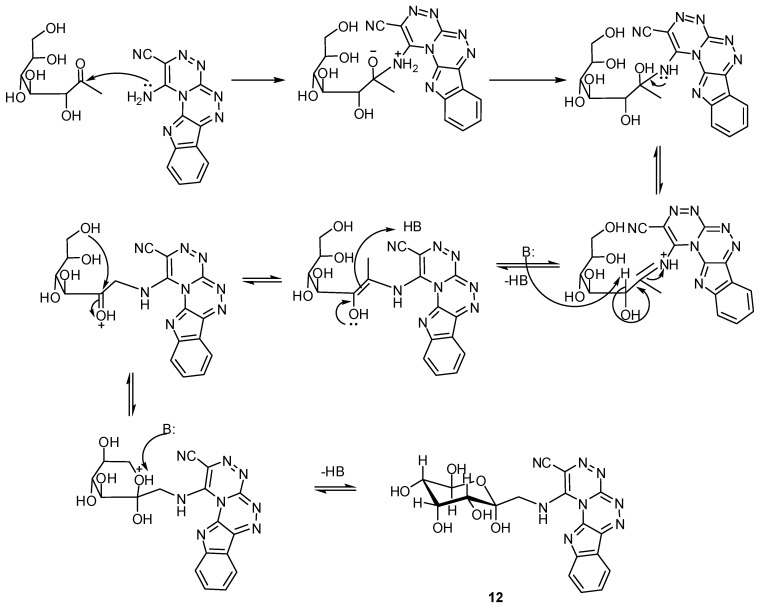
Maillard-type mechanism for the reaction of compound **4** with glucose.

The product of the reaction of glucose with compound **4** varied depending on the solvent used in the reaction, whereby when the reaction carried out in *tert*-butanol the reaction followed the Maillard reaction pathway and the resulting compound was 1-(β*-*d-glucopyranosyl-methylamino)[1,2,4]-triazino[3′,4′:3,4][1,2,4]triazino[5,6-*b*]indole-2-carbonitrile (**12**), while, when the reaction was performed in hot acetic acid the main product is the Schiff base type product, 1-(2,3,4,5,6-pentahydroxyhexylideneamino)[1,2,4]triazino[3′,4′:3,4][1,2,4]triazino[5,6-*b*]indole-2-carbonitrile (**13**). The ^1^H-NMR spectra differentiate between the two compounds, whereby compound **12** showed two signals at δ = 3.93 and 5.12 ppm due to CH_2_ and NH groups, respectively, whereas the Schiff base compound showed an olefinic proton absorption at δ = 7.34 ppm; the ^13^C-NMR also showed a signal at δ = 163.2 ppm in compound **13** corresponding to the HC=N carbon, while, in compound **12** the δ = 52.66 ppm signal is due to a CH_2_ group. The mechanisms illustrating the formation of compounds **12** and **13** are shown in Scheme 4 and Scheme 5.

**Scheme 6:**
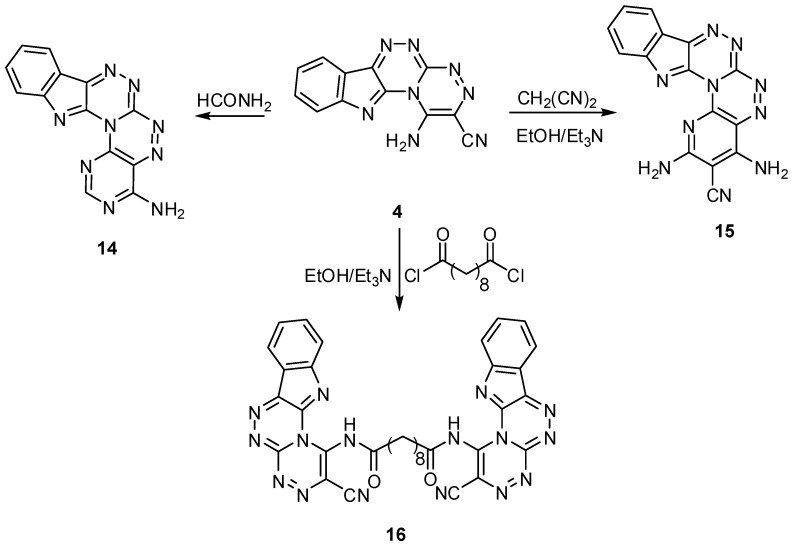
Reactions of 1-amino[1,2,4]triazino[3′,4′:3,4][1,2,4]triazino[5,6-*b*]indole-2-carbonitrile with different reagents.

4-Aminopyrimido[4″,5″:5′,6′][1,2,4]triazino[3′,4′:3,4][1,2,4]triazino[5,6-*b*]indole (**14**) was synthesized by the reaction of compound **4** with formamide, while, the reaction of **4** with malononitrile in basic medium yielded the corresponding 2,4-diaminopyrido[2″,3″:5′,6′][1,2,4]triazino[3′,4′:3,4][1,2,4]triazino[5,6-*b*]indole-3-carbonitrile (**15**). On the other hand, the most amazing compound in this group of products, *N*,*N*′-bis(2-cyano[1,2,4]triazino[3′,4′:3,4]-[1,2,4]triazino[5,6-*b*]indol-1-yl)decanediamide (**16**) was formed from the reaction of **4** with sebacoyl chloride in basic medium [Scheme 6]. The marvelous feature of compound **16** is the anticancer activity it shows against all types of cell lines, more than the standard drug used. The structures of compounds **14**, **15** and **16** are proved using their spectral data (see the Experimental section).

A group of six-ring compounds are synthesized using compound **6** as starting material. The reaction of compound **6** with carbon disulphide in alcoholic KOH under reflux for 9 h gave the cyclic derivative [1,2,4]triazolo[1′′′,5′′′:1′′,6′′]pyrimido[4′′,5′′:5′,6′][1,2,4]triazino[3′,4′:3,4][1,2,4]-triazino-[5,6-*b*]indole-2(1*H*)-thione (**17**) directly and no evidence for the formation of the dithioic acid derivative, The ^1^H-NMR of compound **17** showed no signal due to a SH group with the presence of a signal due to the NH of a triazolo ring at δ = 11.30 ppm. The reaction of compound **17** with hydrazine hydrate in boiling ethanol gave the starting material **6** in poor yield.

**Scheme 7:**
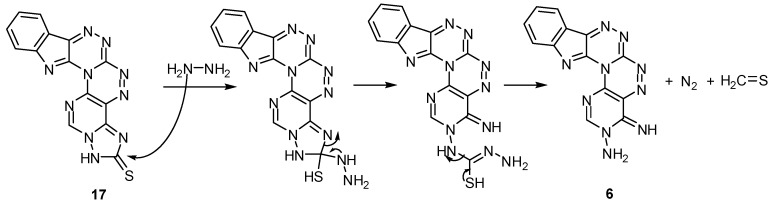
The proposed mechanism for the conversion of compound **17** to its substrate by hydrazine hydrate.

The mechanism proposed for this amazing reaction is illustrated in Scheme 7. Methylation of compound **17** with methyl iodide in sodium acetate yielded the S-methyl derivative **18**. The structure of compound **18** was confirmed by its IR, ^1^H, and ^13^C-NMR data. The IR showed the disappearance of the NH peak at 3266 cm^−1^ and the C=S peak at 1338 cm^−1^ in compound **17**. The ^1^H-NMR of compound **18** showed a new signal due to the SCH_3_ protons at δ = 3.01 ppm and no signal for a NH group. The ^13^C-NMR spectrum also supported the methylation of the S atom by a signal appearing at δ = 13.5 ppm due to the SCH_3_ carbon.

**Scheme 8:**
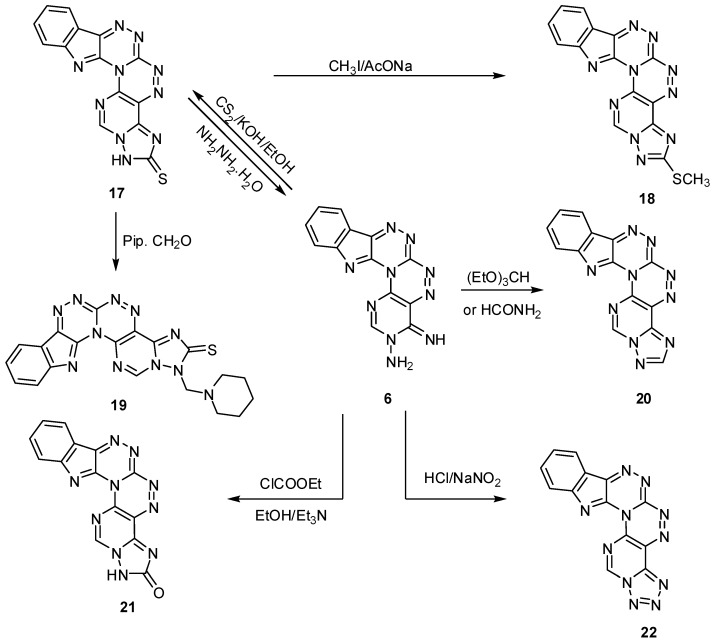
Some reactions on 4-iminopyrimido[4″,5″:5′,6′][1,2,4]triazino[3′,4′:3,4][1,2,4]triazino[5,6-*b*]-indol-3(4H)-amine.

Mannich reaction on compound **6** with formaldehyde and piperidine (Scheme 8) afforded 1-(piperidin-1-ylmethyl)[1,2,4]triazolo[1′′′,5′′′:1′′,6′′]pyrimido[4′′,5′′:5′,6′][1,2,4]triazino[3′,4′:3,4][1,2,4]- triazino[5,6-*b*]indole-2(1*H*)-thione (**19**). The structure of compound **19** was elucidated from its spectral analysis, where the ^1^H-NMR and ^13^C-NMR spectra showed signals due to the piperidine ring hydrogen and carbons at δ = 1.35–2.35 and 24.03, 25.73, 50.23, 51.43 ppm, respectively. Reaction of compound **6** with triethyl orthoformate in boiling DMF yielded [1,2,4]triazolo[1′′′,5′′′:1′′,6′′]pyrimido[4′′,5′′:5′,6′][1,2,4]triazino[3′,4′:3,4][1,2,4]triazino-[5,6-*b*]-indole (**20**). The same product was obtained when compound **6** was reacted with formamide in boiling DMF (Scheme 8). The IR of compound **20** showed no peaks due to NH_2_ or NH groups, and the same observation was obtained from the ^1^H-NMR spectrum. Reaction of ethyl chloroformate with compound **6** in boiling ethanol using Et_3_N as a basic medium formed [1,2,4]triazolo[1′′′,5′′′:1′′,6′′]pyrimido[4′′,5′′:5′,6′][1,2,4]triazino[3′,4′:3,4][1,2,4]-triazino[5,6-*b*]indol-2(1*H*)-one(**21**). On the other hand, diazotization of compound **6** yielded the tetrazolo derivative tetrazolo[1′′′,5′′′:1′′,6′′]pyrimido[4′′,5′′:5′,6′][1,2,4]triazino[3′,4′:3,4][1,2,4]triazino-[5,6-*b*]indole (**22**) (Scheme 8).

The IR of compound **21** showed a peak due to a carbonyl group at 1654 cm^−1^ with disappearance of the NH_2_ and NH peaks, while compound **22** showed no major functional groups in its IR except the C=N group.

## 3. Cytotoxic Activity

**Figure 2:**
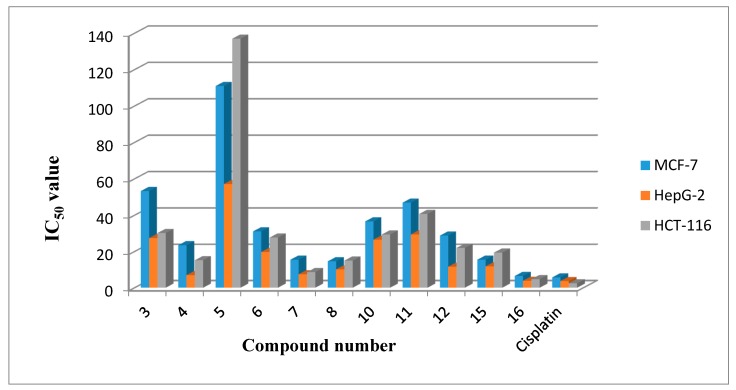
The antitumor activities of the tested compounds expressed as IC_50_ values and compared with reference standard drugs evaluated on breast, liver cancer and colon carcinoma cell lines.

The in vitro growth inhibitory activity of the synthesized compounds was investigated in comparison with a well-known anticancer standard drug (cisplatin) under the same conditions using a colorimetric MTT assay. Data generated were used to plot a dose response curve from which the concentration of test compounds required to kill 50% of cell population (IC_50_) was determined (Figure 2). The results revealed that all the tested compounds showed inhibitory activity to the tested tumor cell lines in a concentration dependent manner. Cytotoxic activity was expressed as the mean IC_50_ of three independent experiments.

Interestingly, the results, presented in Table 1 and Figure 2, show that compound **16** was the most active against the three tested carcinoma cell lines (HepG2, HCT-116 and MCF-7, respectively), giving IC_50_ values of 3.82, 4.73 and 6.52 μg/mL compared with the cisplatin reference drug. 

The order of activity against breast carcinoma cell line (MCF-7) was **16**, **8**, **15**, **7**, **4**, **12**, **6**, **10**, **11**, **3** and **5**, with IC_50_ values of 6.52 ± 0.6, 14.6 ± 1.2, 15.5 ± 0.7, 15.5 ± 0.8, 23.5 ± 0.8, 28.8 ± 0.8, 31.1 ± 0.7, 36.6 ± 1.8, 46.9 ± 3.4, 53.3 ± 1.9, and 111 ± 2.9 μg/mL, respectively. 

The order of activity against the liver carcinoma cell line (HepG2) was **16**, **4**, **7**, **8**, **12**, **15**, **6**, **10**, **3**, **11** and 5, with IC_50_ values of 3.82 ± 0.3, 6.93 ± 0.3, 7.4 ± 0.5, 10.1 ± 0.9, 11.6 ± 0.8, 11.8 ± 0.6, 19.7 ± 0.9, 26.5 ± 0.4, 27.4 ± 0.8, 29.4 ± 0.8, and 57.1 ± 1.4 μg/mL, respectively.

The order of activity against colon carcinoma cell line (HCT-116) was **16**, **7**, **4**, **8**, **15**, **12**, **6**, **10**, **3**, **11** and 5, with IC_50_ values of 4.73 ± 0.7, 8.64 ± 0.8, 15.2 ± 0.4, 15 ± 0.6, 19.4 ± 1.8, 22 ± 0.7, 27.7 ± 0.9, 29.4 ± 0.7, 30.2 ± 0.8, 40.7 ± 2.3, and 137 ± 6.4 μg/mL, respectively.

## 4. Molecular Docking Studies

**Figure 3:**
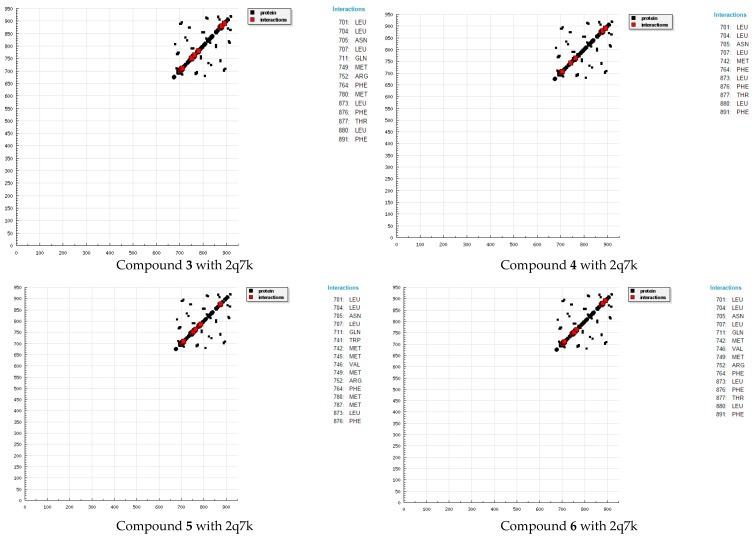

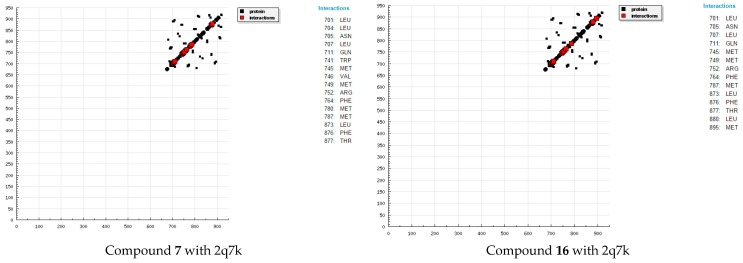
HB plots of interaction between some synthesized compounds and prostate cancer 2q7k hormone.

**Figure 4:**
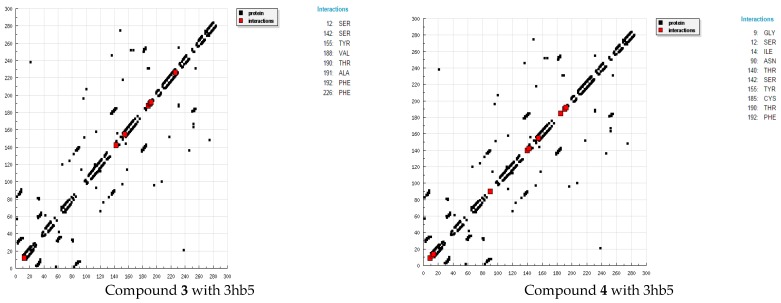

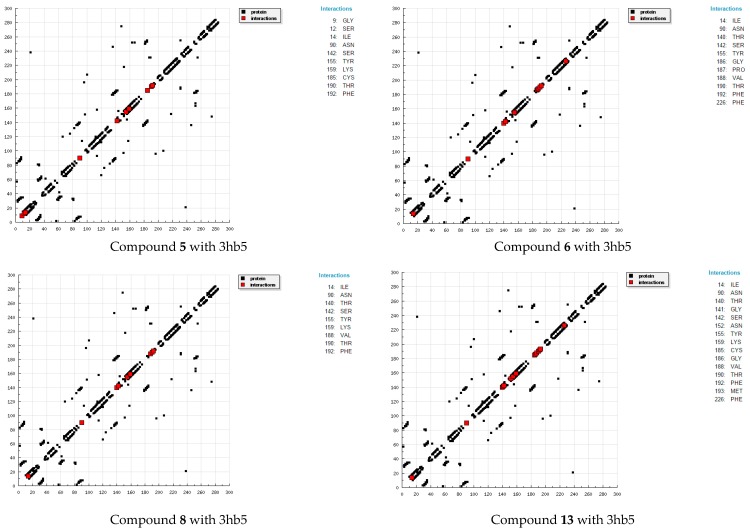
HB plots of interaction between some synthesized compounds and breast ‎cancer (3hb5) hormone.

**Figure 5:**
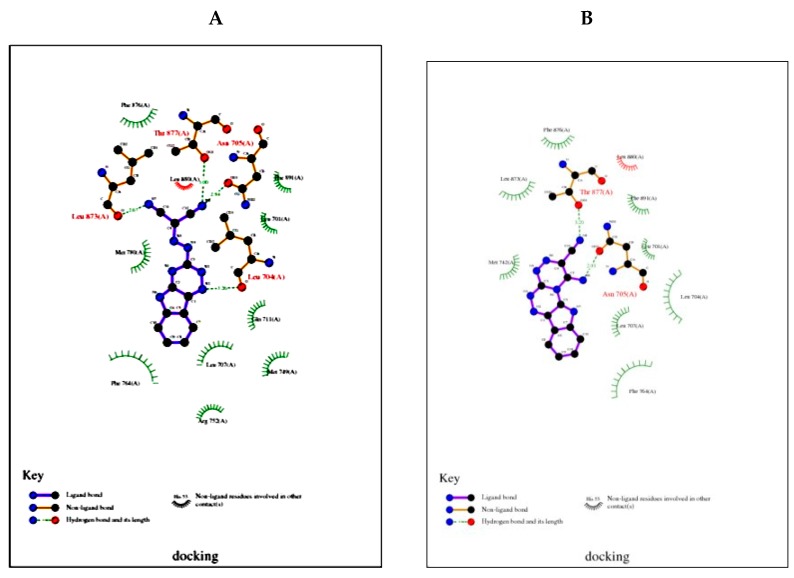
(**A**). 2D plot of interaction between the compound **3** receptor prostate ‎cancer (2q7k); (**B**). 2D plot of interaction between the compound **4** and receptor prostate ‎cancer (2q7k).

**Figure 6:**
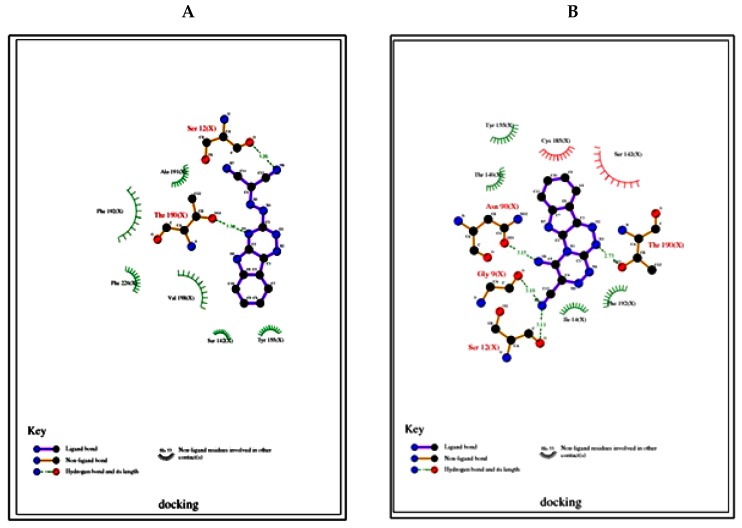
(**A**). 2D plot of interaction between the compound **3** and receptor breast ‎cancer (3hb5); (**B**). 2D plot of interaction between the compound **4** and receptor breast ‎cancer (3hb5).

Molecular docking is a key tool in computer drug design [31]. The focus of molecular docking is to simulate the molecular recognition process. Molecular docking aims to achieve an optimized conformation for both protein and drug with relative orientation between them such that the free energy of the overall system is minimized. The results showed a possible arrangement between free compound as a drugs and receptors (2q7k) and (3hb5). The docking study showed a favorable interaction between the compounds and the receptor, and the calculated energy is presented in Table 2 and Table 3. According to our results (Figure 3 and Figure 4), HB plots indicate that the drug compounds bind to the 2q7k and 3hb5 proteins with hydrogen bond interactions and there are decomposed interaction energies of synthesized compounds with 2q7k and 3hb5 (Table 4 and Table 5). The calculated efficiency is favorable. K_i_ values (estimated using AutoDock) were compared with experimental K_i_ values, when available, and the Gibbs free energy is negative. Also, based on these data, we can propose that an interaction between the 2q7k and 3hb5 receptors and our compounds is possible. The 2D plots of interaction between the compound **3** and compound **4** and prostate cancer receptor (2q7k) and receptor breast cancer enzymes are shown in Figure 5 and Figure 6. From an analysis of the values, it is evident that the binding energy of our compounds decreases. Binding energies are the most widely used mode of measuring the binding affinity of a compound. Thus, a decrease in binding energy due to mutation will increase the binding affinity of the compounds towards the receptor. The characteristic feature of the compounds is represented by the presence of several active sites available for hydrogen bonding. This feature gives them the ability to be good binding inhibitors to the protein and will help to produce augmented inhibitory compounds. The results confirm that the fused triazines as a drug compounds are efficient, preventing prostate cancer 2q7k and breast ‎cancer 3hb5 hormone effectively, which inhibits assembly. This interaction could activate apoptosis in cancer cells for interactions with synthesized compounds. Binding energies are most widely used as a mode of measuring the binding affinity of compounds. Thus, a decrease in binding energy due to mutation will increase the binding affinity of the compounds towards the receptor [25,26,27,28,29]. The characteristic feature of compounds was the presence of several active sites available for hydrogen bonding. This software includes code developed by the Theoretical and Computational Biophysics Group in the Beckman Institute for Advanced Science and Technology at the University of Illinois at Urbana-Champaign [31,32,33,34].

## 5. Materials and Methods

### 5.1. General Information

All chemicals were purchased from Sigma-Aldrich (Taufkirchen, Germany). The melting points were measured by a digital Electrothermal IA 9100 Series apparatus Cole-Parmer, Beacon Road, Stone, Staffordshire, ST15 OSA, UK) and were uncorrected. IR spectra were recorded on an ATRAlpha FTIR spectrophotometer (Billerica, MA, USA) from 400 to 4000 cm^−1^. ^1^H-NMR and ^13^C-NMR spectra were recorded on an AC-850 Hz instrument (Bruker, Billerica, Massachusetts). Chemical shifts were expressed as (ppm) relative to TMS as an internal standard, and DMSO-d6 was used as the solvent. Mass spectra were recorded on a GC-MS-QP 1000 EX spectrometer (Shimadzu, Kyoto, Japan). The pharmacological study was carried out at Al-Azhar University, The Regional Center for Mycology & Biotechnology, Elemental analyses were performed at the Micro-analytical Center of Cairo University.

*[4H-[1,2,4]Triazino[5,6-b]indol-3-yldiazenyl]malononitrile* (**3**). 3-Amino-4*H*-[1,2,4]triazino[5,6-*b*]indole (1.85 g, 0.01 mol) was dissolved in conc. HCl (6 mL) and cooled to 4 °C, then NaNO_2_ (0.7 g, 0.01 mol) dissolved in H_2_O (5 mL) was added to the previous solution, and the prepared mixture was added to a stirred cold solution of malononitrile (0.66 g, 0.01 mol) and sodium acetate (2 g) in H_2_O (15 mL). After stirring 15 min a yellow precipitate was formed, filtered off and washed with water, dried and washed with ethanol, yield, 85%, m.p.: 278–280 °C. IR: 3201 cm^−1^ (NH), 2210, 2217 cm^−1^ (2CN) and 1618 cm^−1^ (C=N). ^1^H-NMR (DMSO-*d*_6_): δ = 6.93 (s, H, CH(CN)_2_), 7.01 (dd, 1H, C_8_H), 7.92 (d, 1H, *J =* 15.6 Hz, C_9_H), 7.32 (dd, 1H, C_7_H), 7.70 (d, 1H, *J =* 15.0 Hz, C_6_H), 11.42 (s, 1H, NH). ^13^C-NMR (DMSO-*d*_6_): δ = 73.51 (CH), 110.6 (CN), 119.4, 120.7, 121.6, 122.3, 126.5, 132.0, 137.0, 142.9, 156.0, and 161.7 (Ar-C, C=C and C=N). Anal. Calcd.for C_12_H_6_N_8_ (262.23): C, 54.96; H, 2.31; N, 42.73; Found: C, 54.79; H, 2.12; N, 42.65. LCMS (ESI) *m*/*z* (int. %) (263 (9.1)) (M + H)^+^; calcd. for (C_12_H_6_N_8_) (262.23): 262 (82.8), 195 (78.9), 168 (100), 140 (47.4), 113 (44.6), 71 (54.6), 69 (48.9), 57 (100), 55 (77.9).

*1-Amino[1,2,4]triazino[3′,4′:3,4][1,2,4]triazino[5,6-b]indole-2-carbonitrile* (**4**). Compound **3** (2.62 g, 0.01 mol) was refluxed in ethanol (20 mL) for 3 h during which the yellow precipitate was converted into red crystals, which were filtered off and washed with ethanol. Yield, 94%, m.p.: 310–312 °C. IR: 3351–3313 cm^−1^ (NH_2_), 2209 cm^−1^ (CN) and 1623 cm^−1^ (C=N). ^1^H-NMR (DMSO-*d*_6_): δ = 6.90 (s, 2H, NH_2_), 7.09 (dd, 1H, C_8_H), 7.52 (dd, 1H, C_7_H), 7.57 (d, 1H, *J =* 12.4 Hz, C_9_H), 7.84 (d, 1H, *J =* 15.6 Hz, C_6_H). ^13^C-NMR (DMSO-*d*_6_): δ = 111.5 (CN), 111.6, 113.0, 118.6, 121.2, 122.9, 125.8, 137.8, 146.5, 150.5, 152.1, and 163.7 (Ar-C, C=C and C=N). Anal. Calcd. for C_12_H_6_N_8_ (262.23): C, 54.96; H, 2.31; N, 42.73; Found: C, 54.81; H, 2.21; N, 42.69. LCMS (ESI) *m*/*z* (int. %) (263 (2.9)) (M + H)^+^; calcd. for [(C_12_H_6_N_8_) (262.23)], 262 (82.8), 195 (100), 169 (49.4), 168 (66.4), 167 (41.6), 140 (61.6), 115 (20.5), 113 (43.3), 87 (25.5), 62 (23.1), 51 (10.9), 50 (13.3).

*Ethyl (2-cyano[1,2,4]triazino[3',4':3,4][1,2,4]triazino[5,6-b]indol-1-yl)imidoformate* (**5**). A solution of compound **4** (2.62 g, 0.01 mol) in triethyl orthoformate (10 mL) was refluxed for 1 h, and the yellow-orange powder that was formed was filtered, dried, and crystallized from methanol. Yield, 62%, m.p.: 217–219 °C. IR: 3001–2890 cm^−1^ (CH), 2211 cm^−1^ (CN) and 1620 cm^−1^ (C=N). ^1^H-NMR (DMSO-*d*_6_): δ = 1.26 (t, 3H, *J* = 7.1 Hz, CH_3_), 3.51 (q, 2H, *J* = 12.4 Hz, CH_2_), 7.11 (dd, 1H, C_8_H), 7.54 (dd, 1H, C_7_H), 7.58 (d, 1H, *J =* 12.8 Hz, C_9_H), 7.85 (d, 1H, *J =* 15.4 Hz, C_6_H). ^13^C-NMR (DMSO-*d*_6_): δ = 13.77 (CH_3_), 63.02 (CH_2_), 119.3 (CN), 120.2, 120.8, 121.1, 128.6, 132.6, 132.8, 140.6, 149.3, 151.9, 159.8, 160.2 and 162.5 (Ar-C, C=C and C=N). Anal. Calcd. for C_15_H_10_N_8_O (318.29): C, 56.60; H, 3.17; N, 35.20; Found: C, 56.40; H, 3.02; N, 35.13. LCMS (ESI) *m*/*z* (int. %): (318 (52.1)) (M + H)^+^; calcd. for [(C_15_H_10_N_8_O) (318.29)]:, 203 (30.7), 175 (92.5), 161 (16.9), 133 (23.4), 132 (24.9), 119 (17.9), 105 (25.6), 104 (100), 103 (22.0), 90 (17.4), 78 (21.0), 77 (37.7), 76 (28.3), 65 (12.4), 64 (9.8), 63 (12.4), 58 (14.7), 51 (20.6), 50 (15.6).

*3(4H)-Amino-4-iminopyrimido[4″,5″:5′,6′][1,2,4]triazino[3',4':3,4][1,2,4]triazino[5,6-b]indole* (**6**)*.* A mixture of compound **5** (3.18 g, 0.01 mol) and hydrazine hydrate (3 mL (excess), 80%) in DMF (10 mL) was refluxed for 4 h. A yellow precipitate that formed after cooling which filtered off, dried, and crystallized from DMF. Yield, 54%, m.p.: 298–300 °C. IR: 3381–3174 cm^−1^ (NH_2_, H), and 1621 cm^−1^ (C=N). ^1^H-NMR (DMSO-*d*_6_): δ = 5.83 (s, 2H, NH_2_), 7.09 (dd, 1H, *C*_8_H), 7.52 (dd, 1H, C_7_H), 7.57 (d, 1H, *J =* 12.8 Hz, C_9_H), 7.83 (d, 1H, *J =* 15.4 Hz, C_6_H), 10.13 (s, 1H, NH). ^13^C-NMR (DMSO-*d*_6_): δ = 120.2, 120.8, 121.1, 128.6, 132.6, 132.8, 140.6, 145.3, 149.3, 151.9, 159.8, 160.2 and 162.5 (Ar-C, C=C and C=N). Anal. Calcd.for C_13_H_8_N_10_ (304.27): C, 51.32; H, 2.65; N, 46.03; Found: C, 51.13; H, 2.49; N, 46.13. LCMS (ESI) *m*/*z* (int. %): (304 (0.9)) (M + H)^+^; calcd. for [(C_13_H_8_N_10_) (304.27)], 252 (4.4), 224 (1.8), 196 (2.3), 149 (3.1), 141 (1.9), 127 (2.5), 113 (4.1), 111 (3.0), 99 (8.2), 97 (8.1), 96 (2.3), 85 (27.9), 83 (12.5), 82 (4.3), 71 (51.5), 69 (19.2), 57 (100), 56 (17.3), 55 (36.5).

*(2-Cyano[1,2,4]triazino[3',4':3,4][1,2,4]triazino[5,6-b]indol-1-yl)carbamodithioic acid* (**7**). A mixture of compound **4** (2.62 g, 0.01 mol), carbon disulfide (1.0 mL) and potassium hydroxide (0.06 g, 0.01 mol) in ethanol (20 mL) was heated under reflux for 3 h. After cooling, the mixture was poured onto water and then acidified with dilute HCl. The red solid obtained was filtered off, dried, and then crystallized from ethanol to give red crystals. Yield, 85%, m.p.: 307–309 °C. IR: 3287 cm^−1^ (NH), 2589 cm^−1^ (SH), 2209 cm^−1^ (CN), 1618 cm^−1^ (C=N), and 1340 cm^−1^ (C=S). ^1^H-NMR (DMSO-*d*_6_): δ = 7.12 (dd, 1H, C_8_H), 7.57 (dd, 1H, C_7_H), 7.59 (d, 1H, *J =* 12.8 Hz, C_9_H), 7.82 (d, 1H, *J =* 15.4 Hz, C_6_H), 10.86 (s, 1H, NH), 13.98 (s, 1H, SH).^13^C-NMR (DMSO-*d*_6_): δ = 117.3 (CN), 119.5, 120.8, 121.3, 125.4, 127.1, 132.0, 136.8, 152.6, 156.8, 159.0, 161.3 and 174.4 (Ar-C, C=C, C=N and C=S). Anal. Calcd.for C_13_H_6_N_8_S_2_ (338.37): C, 46.14; H, 1.79; N, 33.12; S, 18.95; Found: C, 45.95; H, 1.64; N, 33.01; S, 18.77. LCMS (ESI) *m*/*z* (int. %): (338 (9.1)) (M + H)^+^; calcd for [(C_13_H_6_N_8_S_2_) (338.37)], 262 (4.0), 192 (2.3), 157 (11.5), 129 (9.14), 104 (21.8), 103 (32.9), 96 (11.9), 90 (14.8), 88 (13.5), 76 (67.5), 63 (100), 59 (37.9).

*Pyrimido[4″,5″:5',6'][1,2,4]triazino[3',4':3,4][1,2,4]triazino[5,6-b]indol-2,4(1H,3H)-dithione* (**8**)*. Method A*: A mixture of compound **7** (0.34 g, 0.001 mol) in ethanolic sodium ethoxide (20 mL,0.07 g, 0.001 mol) was refluxed for 8 h. After cooling, the mixture was poured onto water and acidified with HCl. The orange yellow solid formed was collected by filtration and then crystallized from dimethylformamide to give compound **8**. Yield, 74%, m.p.: over 300 °C. *Method B*: Compound **4** (0.26 g, 0.001 mol) and carbon disulfide (2 mL) in dry pyridine (15 mL) was refluxed for 15 h. After cooling, the mixture was poured onto water and then acidified with HCl. The obtained precipitate was collected by filtration and then crystallized from dimethylformamide to yield **8**. Yield 67%, m.p. over 300 °C. IR: 3392–3347 cm^−1^ (2NH), 1623 cm^−1^ (C=N), and 1351–1250 cm^−1^ (2C=S). ^1^H-NMR (DMSO-*d*_6_): 6.93 (d, 1H, *J =* 15.0 Hz, C_9_H), 7.01 (dd, 1H, C_8_H), 7.31 (dd, 1H, C_7_H), 7.69 (d, 1H, *J =* 14.4 Hz, C_6_H), 11.40 (s, 1H, NH), 12.50 (s, 1H, NH).^13^C-NMR (DMSO-*d*_6_): δ = 110.9, 119.5, 120..8, 121.7, 122.4, 126.6, 132.0, 133..3, 137.0, 142.9, 156.0, 161.8, and 164.0 (Ar-C, C=C, C=N and C=S). Anal. Calcd. for C_13_H_6_N_8_S_2_ (338.37): C, 46.14; H, 1.79; N, 33.12; S, 18.95; Found: C, 46.01; H, 1.69; N, 32.94; S, 18.78. 

LCMS (ESI) *m*/*z* (int. %) (338 (26.5)) (M + H)^+^; calcd. for (C_13_H_6_N_8_S_2_) (338.37), 203 (25.5), 175 (82.3), 161 (14.8), 133 (21.3), 132 (20.5), 119 (16.2), 118 (12.2), 105 (24.8), 104 (100), 90 (17.4), 78 (23.0), 77 (42.4), 76 (32.4), 75 (12.5), 64 (11.8), 63 (14.8), 58 (16.8), 57 (11.9), 51 (26.1), 50 (19.2).

*2,4-Diaminopyrimido[4″,5″:5',6'][1,2,4]triazino[3',4':3,4][1,2,4]triazino[5,6-b]indole* (**9**). A mixture of **4** (0.26 g, 0.001 mol) and urea and/or thiourea (0.001 mol) was refluxed in glacial AcOH (20 mL) containing HCl (1 mL) for 3 h. After cooling the reaction mixture was poured onto cold water and then neutralized with ammonia solution. The precipitate formed was filtered off and then crystallized from methanol to give orange-red crystals. Yield, 57% from urea and 72% from thiourea, m.p.: over 300 °C. IR: 3413–3761 cm^−1^ (2NH_2_), and 1620 cm^−1^ (C=N). ^1^H-NMR (DMSO-*d*_6_): δ = 5.55 (s, 2H, NH_2_), 5.67 (s, 2H, NH_2_), 6.94 (d, 1H, *J =* 15.6 Hz, C_9_H), 7.02 (dd, 1H, *C*_8_H), 7.28 (dd, 1H, C_7_H), 7.70 (d, 1H, *J =* 14.4 Hz, C_6_H).^13^C-NMR (DMSO-*d*_6_): δ = 110.9, 111.2, 119.5, 120.6, 121.7, 122.4, 132.1, 137.0, 142.9, 156.0, 157.3, 160.8, and 161.8 (Ar-C, C=C, and C=N). Anal. Calcd. for C_13_H_8_N_10_ (304.27): C, 51.32; H, 2.65; N, 46.03; Found: C, 51.18; H, 2.56; N, 45.89. LCMS (ESI) *m*/*z* (int. %) (304 (76.5)) (M + H)^+^; calcd. for (C_13_H_8_N_10_) (304.27), 203 (16.8), 175 (42.3), 157 (6.95), 146 (3.48), 133 (11.9), 132 (12.6), 119 (10.44), 104 (57.9), 103 (20.7), 90 (11.6), 77 (29.5), 76 (100), 63 (11.2), 59 (17.9), 51 (16.6).

*3-Amino-1H-pyrazolo[3″,4″:5',6'][1,2,4]triazino[3',4':3,4][1,2,4]triazino[5,6-b]indole* (**10**). Compound **4** (0.26 g, 0.001 mol) was refluxed for 2 h with hydroxylamine hydrochloride (0.007 g, 0.001 mol) in glacial acetic acid ( 20 mL) containing anhydrous sodium acetate (0.08 g, 0.001 mol) as a catalyst. After cooling the reaction mixture was poured onto cold water to give an orange-yellow precipitate which filtered, dried and crystallized from ethanol. Yield, 81%, m.p.: 297–299 °C. IR: 3392–3754 cm^−1^ (NH_2_), and 1621 cm^−1^ (C=N). ^1^H-NMR (DMSO-*d*_6_): δ = 6.10 (s, 2H, NH_2_), 6.92 (d, 1H, *J =* 15.0 Hz, C_9_H), 7.02 (dd, 1H, C_8_H), 7.30 (dd, 1H, C_7_H), 7.68 (d, 1H, *J =* 14.4 Hz, C_6_H), 12.33 (s, 1H, NH). ^13^C-NMR (DMSO-*d*_6_): δ = 117.9, 121.4, 121.7, 128.3, 132.8, 135.2, 139.4, 139.7, 140.5, 149.3, 159.1 and 159.9 (Ar-C, C=C, and C=N). Anal. Calcd. for C_12_H_7_N_9_ (277.24): C, 51.99; H, 2.54; N, 45.47; Found: C, 52.12; H, 2.50; N, 45.32. LCMS (ESI) *m*/*z* (int. %) (277 (53.5)) (M + H)^+^; calcd. for (C_12_H_7_N_9_) (277.24), 203 (30.4), 176 (10.3), 175 (100), 161 (21.1), 133 (18.7), 132 (20.5), 119 (12.2), 105 (16.8), 104 (63.7), 103 (12.7), 90 (11.2), 78 (13.3), 77 (23.7), 76 (16.4), 51 (12.9).

*2-Aminopyrimido[4″,5″:5',6'][1,2,4]triazino[3',4':3,4][1,2,4]triazino[5,6-b]indol-4(3H)-thione* (**11**). A mixture of compound **4** (0.26 g, 0.001 mol) and ammonium thiocyanate (0.23 g, 0.003 mol) in acetic acid (20 mL) was refluxed for 9 h. After cooling, the mixture was poured onto cold water; the solid thus separated was filtered off, dried, and crystallized from ethanol. Yield, 74%, m.p.: over 286–288 °C. IR: 3401–3811 cm^−1^ (NH_2_), 1621 cm^−1^ (C=N) and 1349 cm^−1^ (C=S). ^1^H-NMR (DMSO-*d*_6_): δ = 6.93 (s, 2H, NH_2_), 6.91 (d, 1H, *J =* 15.6 Hz, C_9_H), 7.02 (dd, 1H, C_8_H), 7.32 (dd, 1H, C_7_H), 7.68 (d, 1H, *J =* 14.4 Hz, C_6_H), 11.12 (s, 1H, NH). ^13^C-NMR (DMSO-*d*_6_): δ = 117.9, 121.4, 121.7, 128.3, 132.8, 135.2, 139.4, 139.7, 140.5, 149.3, 159.1 and 159.9 (Ar-C, C=C, and C=N). Anal. Calcd. for C_13_H_7_N_9_S (321.32): C, 48.59; H, 2.20; N, 39.23; S, 9.98; Found: C, 48.42; H, 2.07; N, 39.11; S, 9.78. LCMS (ESI) *m*/*z* (int. %) (321 (56.4) (M + H)^+^; calcd for (C_13_H_7_N_9_S) (321.32), 203 (22.7), 175 (66.5), 161 (11.7), 133 (17.7), 132 (17.1), 119 (13.8), 118 (11.3), 104 (100), 103 (25.4), 91 (10.0), 90 (20.4), 77 (51.2), 76 (42.2), 63 (18.8), 59 (22.8), 58 (20.6), 50 (25.9).

*1-(β-d-Glucopyranosymethylamino)[1,2,4]triazino[3',4':3,4][1,2,4]triazino[5,6-b]indol-2-carbonitrile* (**12**). A mixture of compound **4** (0.26 g, 0.001 mol) and glucose (0.18 g, 0.001 mol) in *tert-*butanol (15 mL) was refluxed for 5 h. After cooling, the formed yellow-orange crystals were filtered off, dried, and crystallized from ethanol. Yield, 71%, m.p.: 281–283 °C. IR: 3439 cm^−1^ (OH), 3210 m^−1^ (NH), 3011–2859 cm^−1^ (CH, aromatic and aliphatic), 2210 cm^−1^ (CN), 1621 cm^−1^ (C=N). ^1^H-NMR (DMSO-*d*_6_): δ = 3.32–4.99 (m, 4H, 4CH), 3.93 (s, 2H, CH_2_), 4.01 (s, 1H, NH), 4.62–4.78 (m, 5H, 5OH), 6.95 (d, 1H, *J =* 16.2 Hz, C_9_H), 7.03 (dd, 1H, C_8_H), 7.33 (dd, 1H, C_7_H), 7.70 (d, 1H, *J =* 14.4 Hz, C_6_H), 5.12 (s, 1H, NH).. ^13^C-NMR (DMSO-*d*_6_): δ = 52.66 (CH_2_), 71.61, 72.00, 75.90, 93.55, 101.2 (sp^3^ carbons, CHOH,), 111.0, 119.32 (CN), 120.7, 121.8, 125.3, 127.3, 132.9, 136.2, 145.2, 152.3, 156.2, and 161.1 (Ar-C, C=C, and C=N). Anal. Calcd. for C_18_H_16_N_8_O_6_ (440.37): C, 49.09; H, 3.66; N, 25.45; Found: C, 48.91; H, 3.51; N, 25.40.

*1-(2,3,4,5,6-Pentahydroxyhexylideneamino)[1,2,4]triazino[3',4':3,4][1,2,4]triazino[5,6-b]indol-2-carbonitrile* (**13**). A mixture of **4** (0.26 g, 0.001 mol) and glucose (0.18 g, 0.001 mol) in acetic acid/water (15/5 mL) was refluxed for 8 h. After cooling, the resulting solution was poured onto cold water. The red solution was extracted with CH_2_Cl_2_ (30 mL). After evaporation of the CH_2_Cl_2_ a pale red solid resulted which was filtered off, dried, and crystallized from ethanol. Yield, 52%, m.p.: 268–270 °C. IR: 3432 cm^−1^ (OH), 2992–2860 cm^−1^ (CH, aromatic and aliphatic), 2208 cm^−1^ (CN), 1618 cm^−1^ (C=N). ^1^H-NMR (DMSO-*d*_6_): δ = 3.44–5.11 (m, 4H, 4CH), 4.73–4.93 (m, 2H, CH_2_), 4.30–4.81 (m, 5H, 5OH), 6.94 (d, 1H, *J =* 15.0 Hz, C_9_H), 7.03 (dd, 1H, C_8_H), 7.31 (dd, 1H, C_7_H), 7.34 (d, 1H, CH=N), 7.70 (d, 1H, *J =* 14.4 Hz, C_6_H). ^13^C-NMR (DMSO-*d*_6_): δ = 63.51, 69.21, 69.58, 71.17, 72.63 (sp^3^ carbons, CHOH, CH_2_), 122.6 (CN), 122.7, 1123.8, 125.4, 127.2, 133.3, 135.2, 140.5, 155.0, 155.6, 160.2, 163.2 and 164.3 (Ar-C, C=C, and C=N). Anal. Calcd. for C_18_H_16_N_8_O_5_ (424.37): C, 50.94; H, 3.80; N, 26.40; Found: C, 50.81; H, 3.67; N, 26.33. 

*4-Aminopyrimido[4″,5″:5',6'][1,2,4]triazino[3',4':3,4][1,2,4]triazino[5,6-b]indole* (**14**). Compound **4** (0.26 g, 0.001 mol) was refluxed for 3 h in formamide (10 mL). After cooling, a reddish brown precipitate was formed. The precipitate was collected by filtration, dried, and crystallized from ethanol. Yield, 41%, m.p.: over 300 °C. IR: 3392–3352 cm^−1^ (NH_2_), 1619 cm^−1^ (C=N). ^1^H-NMR (DMSO-*d*_6_): δ = 6.81 (s, 2H, NH_2_), 6.91 (d, 1H, *J =* 15.6 Hz, C_9_H), 7.02 (dd, 1H, C_8_H), 7.32 (dd, 1H, C_7_H), 7.68 (d, 1H, *J =* 14.4 Hz, C_6_H). ^13^C-NMR (DMSO-*d*_6_): δ = 117.2, 120.6, 121.3, 12701, 129.6, 132.8, 139.6, 146.2, 156.4, 156.7, 159.8, 160.3 and 162.7 (Ar-C, C=C, and C=N). Anal. Calcd. for C_13_H_7_N_9_ (289.25): C, 53.98; H, 2.44; N, 43.58; Found: C, 53.72; H, 2.34; N, 43.41.

*2,4-Diaminopyrido[2″,3″:5',6'][1,2,4]triazino[3',4':3,4][1,2,4]triazino[5,6-b]indol-3-carbonitrile* (**15**). A suspension of compound **4** (0.26 g, 0.001 mol) in ethanol (20 mL) was treated with malononitrile (0.06 g, 0.001 mol) with triethylamine as a catalyst. The mixture was refluxed for 10 h. After cooling, an orange precipitate formed which was filtered off and dried. Crystallization from dioxane resulted compound **15**. Yield, 81%, m.p.: over 300 °C. IR: 3395–3355 cm^−1^ (2NH_2_), 2203 cm^−1^ (CN), 1622 cm^−1^ (C=N). ^1^H-NMR (DMSO-*d*_6_): δ = 6.10 (s, 2H, NH_2_), 6.59 (s, 2H, NH_2_), 6.93 (d, 1H, *J =* 15.6 Hz, C_9_H), 7.03 (dd, 1H, C_8_H), 7.31 (dd, 1H, C_7_H), 7.70 (d, 1H, *J =* 14.4 Hz, C_6_H). ^13^C-NMR (DMSO-*d*_6_): δ = 75.23, 115.6 (CN), 117.6, 120.3, 121.8, 127.5, 127.9, 132.5, 139.3, 145.1, 156.1, 159.4, 162.5, 165.2 and 165.9 (Ar-C, C=C, and C=N). Anal. Calcd.for C_15_H_8_N_10_ (328.29): C, 54.88; H, 2.46; N, 42.67; Found: C, 54.77; H, 2.31; N, 42.52. LCMS (ESI) *m*/*z* (int. %) (328 (67.2)) (M + H)^+^; calcd. for (C_15_H_8_N_10_) (328.29), 309 (43.6), 308 (26.9), 294 (16.4), 111 (10.5), 104 (10.1), 97 (22.5), 96 (11.0), 95 (16.6), 87 (10.0), 85 (26.5), 83 (33.6), 81 (22.1), 76 (22.1), 73 (27.5), 67 (23.6), 60 (25.0), 59 (21.7), 57 (100), 56 (24.9), 55 (78.7), 51 (13.1), 50 (16.4).

*N,N'-bis(2-Cyano[1,2,4]triazino[3',4':3,4][1,2,4]triazino[5,6-b]indol-1-yl)decanediamide* (**16**). A mixture of **4** (0.26 g, 0.001 mol), sebacoyl chloride (0.24 g, 0.21 mL, 1 mmol) and a catalytic amount of TEA in DMF (20 mL) was refluxed for 5 h. The reaction was cooled and poured onto ice water. The solid was filtered off and crystallized from ethanol to result in **16** as orange-yellow crystals. Yield 67%, m.p. 284–286 °C. IR: 3271–3284 cm^−1^ (2NH), 2203–2210 cm^−1^ (2CN), 1674–1661 cm^−1^ (2C=O), 1624 cm^−1^ (C=N). ^1^H-NMR (DMSO-*d*_6_): δ = 1.01 (m, 4H, 2CH_2_), 1.13 (m, 4H, 2CH_2_), 2.20 (m, 4H, 2CH_2_), 3.39 (t, 4H, 2COCH_2_), 6.94 (d, 2H, *J =* 15.6 Hz, 2C_9_H), 7.07 (dd, 2H, 2C_8_H), 7.34 (dd, 2H, 2C_7_H), 7.83 (d, 2H, *J =* 15.6 Hz, 2C_6_H), 11.37 (s, 2H, 2NH). ^13^C-NMR (DMSO-*d*_6_): δ = 25.36, 26.89, 28.40, 35.27 (4CH_2_), 106.7 (CN), 111.7, 119.5, 121.7, 122.8, 125.7, 132.1, 137.8, 146.9, 150.6, 156.0, 161.9 and 163.6 (Ar-C, C=C, and C=N). Anal. Calcd. for C_34_H_26_N_16_O_2_ (690.68): C, 59.13; H, 3.79; N, 32.45; Found: C, 58.93; H, 3.65; N, 32.32.

*[1,2,4]Triazolo[1''',5''':1″,6″]pyrimido[4″,5″:5',6'][1,2,4]triazino[3',4':3,4][1,2,4]triazino[5,6-b]-indol-2(1H)-thione* (**17**). To a stirred solution of compound **6** (0.30 g, 0.001 mol) in ethanol (20 mL), potassium hydroxide (0.06 g, 0.001 mol) and CS_2_ (1.5 mL) were added. The mixture was then refluxed for 9 h and then the solvent evaporated. After that the residue obtained was dissolved in water and neutralized with dil. HCl. The solid thus separated filtered off and crystallized from ethanol to give red crystals. Yield, (86%), m.p.: 297–299 °C. IR: 3266 cm^−1^ (NH), 1624 cm^−1^ (C=N), 1338 cm^−1^ (C=S). ^1^H-NMR (DMSO-*d*_6_): δ = 6.91 (d, 1H, *J =* 15.6 Hz, C_9_H), 7.02 (dd, 1H, C_8_H), 7.32 (dd, 1H, C_7_H), 7.68 (d, 1H, *J =* 14.4 Hz, C_6_H), 9.69 (s, 1H, *CH_pyrimidine_*), 11.30 (s, 1H, NH). ^13^C-NMR (DMSO-*d*_6_): δ = 120.1, 121.8, 122.0, 128.2, 128.5, 140.3, 141.5, 144.9, 147.2, 149.0, 151.3, 160.2, 161.5 and 172.3 (Ar-C, C=C, C=N and C=S). Anal. Calcd. for C_14_H_6_N_10_S (346.33): C, 48.55; H, 1.75; N, 40.44; S, 9.26; Found: C, 48.43; H, 1.66; N, 40.31; S, 9.03.

*2-(Methylthio)[1,2,4]triazolo[1''',5''':1″,6″]pyrimido[4″,5″:5',6'][1,2,4]triazino[3',4':3,4]-[1,2,4]triazino-[5,6-b]indole* (**18**). To a mixture of compound **17** (0.35 g, 0.001 mol) and sodium acetate (1.63 g, 0.02 mol) in ethanol (20 mL) was added the methyl iodide (0.14 g, 0.062 mL, 0.001 mol), then the reaction mixture was refluxed for 5 h. After cooling the solid thus formed was filtered off, washed with water, and crystallized from the ethanol to give yellowish powder of **18**. Yield, (86%), m.p.: 283–285 °C. IR: 3001–2871 cm^−1^ (CH aromatic and aliphatic), 1618 cm^−1^ (C=N). ^1^H-NMR (DMSO-*d*_6_): δ = 3.01 (s, 3H, SCH_3_), 6.93 (d, 1H, *J =* 15.6 Hz, C_9_H), 7.04 (dd, 1H, C_8_H), 7.33 (dd, 1H, C_7_H), 7.71 (d, 1H, *J =* 14.4 Hz, C_6_H), 9.62 (s, 1H, *CH_pyrimidine_*). ^13^C-NMR (DMSO-*d*_6_): δ = 13.5 (SCH_3_), 120.6, 121.7, 122.2, 128.1, 128.9, 132.8, 137.8, 140.4, 141.1, 149.4, 151.8, 160.0, 161.6 and 162.0 (Ar-C, C=C, C=N and C-S). Anal. Calcd. for C_15_H_8_N_10_S (360.36): C, 50.00; H, 2.24; N, 38.87; S, 8.90; Found: C, 49.86; H, 2.16; N, 38.77; S, 8.62.

*1-(Piperidin-1-ylmethyl)[1,2,4]triazolo[1''',5''':1″,6″]pyrimido[4″,5″:5',6'][1,2,4]triazino-[3',4':3,4][1,2,4]- triazino[5,6-b]indol-2(1H)-thione* (**19**). A mixture of **17** (0.35 g, 0.001 mol), aqueous formaldehyde (36%, 1 mL), methanol (25 mL) and piperidine (0.085 g, 0.1 mL, 0.001 mol) was stirred at room temperature for about 3 h. The solid product separated was filtered off and crystallized from dioxane. Yield, (53%), m.p.: over 300 °C. IR: 2994–2841 cm^−1^ (CH aromatic and CH aliphatic), 1624 cm^−1^ (C=N), 1347 cm^−1^ (C=S). ^1^H-NMR (DMSO-*d*_6_): δ =1.35–2.35 (m, 10H, piperidine protons), 5.50 (s, 2H, N-CH_2_), 6.91 (d, 1H, *J =* 15.6 Hz, C_9_H), 7.03 (dd, 1H, C_8_H), 7.31 (dd, 1H, C_7_H), 7.68 (d, 1H, *J =* 14.4 Hz, C_6_H), 8.62 (s, 1H, *CH_pyrimidine_*). ^13^C-NMR (DMSO-*d*_6_): δ = 24.03, 25.73, 50.23, 51.43 (4 CH_2_), 120.1, 121.7, 122.1, 128.9, 132.7, 138.3, 138.9, 140.5, 141.2, 147.6, 149.2, 150.7, 160.0 and 162.0 (Ar-C, C=C, C=N and C=S). Anal. Calcd. for C_20_H_17_N_11_S (443.49): C, 54.16; H, 3.86; N, 34.74; S, 7.23; Found: C, 53.83; H, 3.71; N, 34.66; S, 7.12.

*[1,2,4]Triazolo[1''',5''':1″,6″]pyrimido[4″,5″:5',6'][1,2,4]triazino[3',4':3,4][1,2,4]triazino[5,6-b]indole* (**20**). *Method A*: A mixture of compound **6** (0.30 g, 0.001 mol) and triethyl orthoformate (4 mL) in dimethylformamide (10 mL) was refluxed for 3 h. After cooling the mixture poured onto ice cold water (50 mL), the solid produced was filtered off and crystallized from ethanol to give **19** as yellowish powder. Yield, (54%), m.p.: over 300 °C. *Method B*: A mixture of compound **6** (0.30 g, 0.001 mol) and formamide (5 mL) in dimethylformamide (15 mL) was refluxed for 4 h. After cooling the solution poured onto ice/water (40 mL), the resulted solid was filtered off and crystallized from ethanol to give **19** as yellowish powder. Yield, (46%), m.p.: over 300 °C. IR: 3001–2912 cm^−1^ (CH aromatic), 1622 cm^−1^ (C=N). ^1^H-NMR (DMSO-*d*_6_): δ = 6.92 (d, 1H, *J =* 15.6 Hz, C_9_H), 7.04 (dd, 1H, *C*_8_H), 7.32 (dd, 1H, C_7_H), 7.70 (d, 1H, *J =* 14.4 Hz, C_6_H), 8.41 (s, 1H, *CH_triazole_*), 8.72 (s, 1H, *CH_pyrimidine_*). ^13^C-NMR (DMSO-*d*_6_): δ = 120.1, 121.8, 122.1, 126.2, 128.9, 132.8, 137.1, 139.5, 140.2, 149.2, 151.7, 153.2, 160.0 and 162.6 (Ar-C, C=C, and C=N). Anal. Calcd. for C_14_H_6_N_10_ (314.26): C, 53.51; H, 1.92; N, 44.57; Found: C, 53.35; H, 1.81; N, 44.52.

*[1,2,4]Triazolo[1''',5''':1″,6″]pyrimido[4″,5″:5',6'][1,2,4]triazino[3',4':3,4][1,2,4]triazino[5,6-b]indol-2(1H)-one* (**21**). A mixture of **6** (0.30 g, 0.001 mol) and ethyl chloroformate (0.11 g, 0.001 mol) in DMF (15 mL) and Et_3_N was stirred for 30 min at room temperature, then the mixture refluxed for 3 h, cooled, poured onto ice water and the solid formed was filtered off and crystallized from ethanol. Yield, 64%, m.p.: 291–293 °C. IR: 3276 cm^−1^ (NH), 1654 cm^−1^ (C=O), and 1624 cm^−1^ (C=N). ^1^H-NMR (DMSO-*d*_6_): δ = 6.94 (d, H, *J =* 15.6 Hz, C_9_H), 7.07 (dd, H, C_8_H), 7.34 (dd, H, C_7_H), 7.83 (d, H, *J =* 15.6 Hz, C_6_H), 8.65 (s, 1H, *CH_pyrimidine_*). ^13^C-NMR (DMSO-*d*_6_): δ = 120.1, 121.7, 122.1, 128.7, 132.5, 135.6, 137.5, 140.9, 146.2, 149.2, 149.7, 160.1, 162.4, and 163.9 (Ar-C, C=C, C=N and C=O). Anal. Calcd. for C_14_H_6_N_10_O (330.26): C, 50.91; H, 1.83; N, 42.41; Found: C, 50.64; H, 1.70; N, 42.31.

*Tetrazolo[1''',5''':1″,6″]pyrimido[4″,5″:5',6'][1,2,4]triazino[3',4':3,4][1,2,4]triazino[5,6-b]indole* (**22**). Compound **6** (0.30 g, 0.001 mol) dissolved in acetic acid and conc. HCl mixture (1:1, 8 mL), the solution cooled to 4 °C, sodium nitrite (0.069 g, 0.001 mol, in 5 mL H_2_O) was added and the mixture stirred for 1 h, the solid formed filtered, washed with water, dried, and crystallized from dioxane. Yield, 42%, m.p.: over 300 °C. IR: 1619 cm^−1^ (C=N). ^1^H-NMR (DMSO-*d*_6_): δ = 6.92 (d, H, *J =* 15.6 Hz, C_9_H), 7.03 (dd, H, C_8_H), 7.31 (dd, H, C_7_H), 7.79 (d, H, *J =* 15.6 Hz, *C*_6_H), 8.69 (s, 1H, *CH_pyrimidine_*). ^13^C-NMR (DMSO-*d*_6_): δ = 120.1, 121.7, 122.1, 128.9, 131.6, 132.5, 136.9, 138.2, 140.1, 149.0, 152.8, 160.0, and 162.4 (Ar-C, C=C, C=N and C=O). Anal. Calcd. for C_13_H_5_N_11_ (315.25): C, 49.53; H, 1.60; N, 48.87; Found: C, 49.39; H, 1.48; N, 48.76.

### 5.2. Antitumor Activity Assay

The tested human carcinoma cell lines were obtained from the American Type Culture Collection (ATCC, Rockville, MD, USA). The cells were grown on RPMI-1640 medium supplemented with 10% heat inactivated fetal calf serum, 1% l-glutamine, and 50 µg/mL gentamycin at 37 °C in a humidified atmosphere with 5% CO_2_ incubator (Shel lab 2406, Candler, NC, USA).

For antitumor assays, the tumor cell lines were suspended in medium at concentration 5 × 10^4^ cell/well in Corning^®^ 96-well tissue culture plates, then incubated for 24 h. The tested compounds were then added into 96-well plates (three replicates) to achieve ten concentrations for each compound (started from 500 to 1 µg/mL). Six vehicle controls with media or 0.1% DMSO were run for each 96 well plate as a control. After incubating for 24 h, the numbers of viable cells were determined by the MTT assay [35]. Briefly, the media was removed from the 96 well plate and replaced with 100 µL of fresh culture RPMI 1640 medium without phenol red then 10 µL of the 12 mM MTT (3-[4,5-dimethylthiazol-2-yl]-2,5-diphenyltetrazolium bromide (Sigma, Taufkirchen, Germany) was added to each well including the untreated controls. The 96 well plates were then incubated at 37 °C and 5% CO_2_ for 4 h. An 85 µL aliquot of the media was removed from the wells, and 50 µL of DMSO was added to each well and mixed thoroughly with the pipette and incubated at 37° C for 10 min. Then, the optical density was measured at 590 nm with the microplate reader (SunRise, TECAN, Inc., Männedorf, Switzerland) to determine the number of viable cells and the percentage of viability was calculated as [(ODt/ODc)] × 100% where ODt is the mean optical density of wells treated with the tested sample and ODc is the mean optical density of untreated cells. The relation between surviving cells and drug concentration is plotted to get the survival curve of each tumor cell line after treatment with the specified compound. The 50% inhibitory concentration (IC_50_), the concentration required to cause toxic effects in 50% of intact cells, was estimated from graphic plots of the dose response curve for each conc. using Graphpad Prism software (San Diego, CA, USA) [36].

### 5.3. Theoretical Molecular Docking Techniques

The docking calculations were carried out using Docking Server [32]. The MMFF94 force field [31,32] was used for energy minimization of compound molecules and protein models using Docking Server. Gasteiger partial charges were added to the compound atoms. Non-polar hydrogen atoms were merged, and rotatable bonds were defined. Docking calculations were carried out on 2q7k and 3hb5 protein models. Essential hydrogen atoms, Kollman united atom type charges and solvation parameters were added with the aid of AutoDock tools [27]. Affinity (grid) maps of 20 μg/mL × 20 μg/mL × 20 A° grid points and 0.375 A° spacing were generated using the Autogrid program [34]. AutoDock parameter set- and distance-dependent dielectric functions were used in the calculation of van der Waals and electrostatic terms, respectively. Docking simulations were performed using the Lamarckian genetic algorithm and the Solis and Wets local search method [37,38,39,40]. Initial position, orientation and torsions of the compound molecules were set randomly. Each docking experiment was derived from 10 different runs that were set to terminate after a maximum of 250,000 energy evaluations. The population size was set to 150. During the search, a translation step of 0.2 A° and quaternion and torsion steps of 5 were applied.

## 6. Conclusions

In conclusion, the anticancer screening showed not all the tested compounds exhibited a good result, except compound **16** which exhibited good cytotoxic activities against the three tested carcinoma cell lines (HepG2, MCF-7, and HCT-116) compared with the reference drug cisplatin. This result confirms that increasing of the compound volume is not necessarily effective in producing more active anticancer compounds. The effectiveness of compound **16** may be due to the octa-CH_2_ chain separating two bulky five-ring compounds. Molecular docking modeling was performed on these studied fused triazino compounds against the receptor of prostate cancer 2q7k and breast ‎cancer 3hb5 to get a chance to compare between theoretical and practical results. 

## Figures and Tables

**Table 1:** The antitumor activities of the tested compounds expressed as IC_50_ values and compared with reference standard drugs evaluated on breast, liver cancer and colon carcinoma cell lines

Compound Number	MCF-7	HepG-2	HCT-116
**3**	53.3 ± 1.9	27.4 ± 0.8	30.2 ± 0.7
**4**	23.5 ± 0.8	6.93 ± 0.3	15.2 ± 0.4
**5**	111 ± 2.9	57.1 ± 1.4	137 ± 6.4
**6**	31.1 ± 0.7	19.7 ± 0.9	27.7 ± 0.9
**7**	15.5 ± 0.8	7.4 ± 0.5	8.64 ± 0.8
**8**	14.6 ± 1.2	10.1 ± 0.9	15 ± 0.6
**10**	36.6 ± 1.8	26.5 ± 0.4	29.4 ± 0.7
**11**	46.9 ± 3.4	29.4 ± 0.8	40.7 ± 2.3
**12**	28.8 ± 0.8	11.6 ± 0.8	22 ± 0.7
**15**	15.5 ± 0.7	11.8 ± 0.6	19.4 ± 1.8
**16**	6.52 ± 0.6	3.82 ± 0.3	4.73 ± 0.7
Cisplatin	5.71 ± 0.4	3.67 ± 0.2	2.43 ± 0.2

**Table 2:** Energy values obtained in docking calculations of some synthesized compounds with receptor prostate ‎cancer (2q7k).

Compd.	Est. Free Energy of Binding kcal/mol	Est. Inhibition Constant, K_i_ uM	vdW + Hbond + desolv Energy kcal/mol	Electrostatic Energy kcal/mol	Total Intermolec. Energy kcal/mol	Interact Surface
**3**	−7.04	6.90	−8.22	+0.04	−8.17	509.119
**4**	−7.51	3.12	−8.09	−0.02	−8.11	465.35
**5**	−8.31	8.14	−9.44	−0.04	−9.48	523.761
**6**	−7.98	1.41	−8.03	−0.25	−8.28	508.448
**7**	−8.13	1.09	−9.18	−0.03	−9.21	519.896
**8**	−6.35	22.23	−6.41	+0.06	−6.35	522.705
**12**	−0.96	1.97	−1.87	+0.61	−1.26	583.589
**13**	−0.26	6.47	−7.64	+0.01	−7.63	576.654
**16**	−7.95	1.49	−8.16	−0.40	−8.56	540.988

**Table 3:** Energy values obtained in docking calculations of some synthesized compounds with receptor breast ‎cancer (3hb5).

Compd.	Est. Free Energy of Binding kcal/mol	Est. Inhibition Constant, Ki uM	vdW + Hbond + desolv Energy kcal/mol	Electrostatic Energy kcal/mol	Total Intermolec. Energy kcal/mol	Interact. Surface
**3**	−7.55	2.90	−8.28	−0.18	−8.47	700.877
**4**	−7.66	2.43	−8.03	−0.06	−8.09	639.72
**5**	−7.68	2.34	−8.94	+0.03	−8.91	740.419
**6**	−7.93	1.53	−8.22	−0.01	−8.23	719.146
**9**	−9.31	14.94	−11.12	−0.85	−11.97	1288.56
**12**	−9.15	19.78	−9.76	+0.31	−9.44	851.928
**13**	−8.01	1.35	−10.31	−0.08	−10.39	911.407
**14**	−8.87	31.24	−9.49	−0.59	−10.08	908.153
**15**	−8.74	39.33	−9.08	+0.34	−8.74	846.707

**Table 4:** Decomposed energy of some synthesized compounds with prostate ‎cancer receptor (2q7k).

Compd.	Hydrogen Bonds	Polar	cation-pi	Hydrophobic	Other
**3**	LEU704 (−0.9588) ASN705 (−0.2861) THR877 (0.0967) LEU873 (0.2094)			PHE764 (−0.9244) LEU707 (−0.5332) PHE876 (−0.4595) MET780 (−0.3859) MET749 (−0.3551) LEU701 (−0.1735) LEU880 (0.031)	GLN711 (−0.3009) ARG752 (−0.2282) PHE891 (−0.1823)
**4**	ASN705 (−0.6684) THR877 (−0.4461)			PHE764 (−1.321) LEU873 (−0.6886) LEU707 (−0.4798) LEU701 (−0.2013)	LEU704 (−1.2982) PHE876 (−0.5103) MET742 (−0.4068) PHE891 (−0.3538) LEU880 (0.2352)
**5**	PHE764 (−1.0213) GLN711 (−0.5111)	ARG752 (−0.2593)		LEU704 (−1.3263) LEU873 (−0.8151) LEU707 (−0.6963) MET745 (−0.6501) MET742 (−0.5522) PHE876 (−0.4912) VAL746 (−0.4163 TRP741 (−0.3903 LEU701 (−0.2362) MET780 (−0.0943)	MET749 (−0.5969) ASN705 (−0.5916) MET787 (−0.2392)
**6**	MET742 (0.5327) ASN705 (1.1422)	THR877 (−12.988)	PHE891 (0.4955) PHE876 (1.6611)	PHE764 (−1.6911) MET749 (−0.4918) LEU707 (−0.1047) LEU704 (0.1685) LEU873 (1.6514)	GLN711 (−0.3671) VAL746 (−0.3662) ARG752 (−0.132) LEU880 (0.7122 LEU701 (1.3032)
**7**	GLN711 (−0.2307) MET787 (2.5309)	ARG752 (−0.3853)		LEU873 (−9.3441) PHE764 (−0.6167) MET749 (−0.5857) LEU704 (−0.5848) LEU701 (−0.1264) PHE876 (0.1242) MET780 (0.6784)	MET745 (−0.7229) LEU707 (−0.3795) ASN705 (−0.255) TRP741 (−0.2472) VAL746 (−0.1409 THR877 (0.2176
**8**	LEU704 (−2.7674) ASN705 (21.4001)	THR877 (−1.5585)	PHE876 (−0.703)	PHE764 (−2.7458) LEU701 (−2.0955) LEU873 (−1.5548) MET780 (−0.6025 MET749 (−0.457 VAL746 (1.5949) MET787 (3.0657)	PHE891 (−1.637) LEU880 (−0.6967)
**16**	GLN711 (−0.4793) MET745 (0.1156)	ASN705 (−1.6327) ARG752 (0.3987)		LEU701 (−2.1158) LEU880 (−0.6528) PHE764 (−0.5839) MET749 (−0.3359) PHE876 (1.1185)	LEU873 (−0.4761) LEU707 (−0.4553) MET787 (−0.2292 MET895 (−0.0748) THR877 (1.3407)

**Table 5:** Decomposed energy of some synthesized compounds with breast ‎cancer receptor (3hb5).

Compd.	Hydrogen Bonds	Polar	cation-pi	Hydrophobic	Other
**3**	SER12 (−0.443) THR190 (0.8132)			PHE192 (−1.7843) VAL188 (−1.341) PHE226 (−0.4523)	ALA191 (−0.3955) TYR155 (−0.2826) SER142 (−0.0999)
**4**	ASN90 (−1.0823) SER12 (−0.2674) GLY9 (−0.009) THR190 (0.5483)			ILE14 (−0.9193) CYS185 (1.4843)	TYR155 (−1.2233) PHE192 (−1.1514) THR140 (−0.9162) SER142 (4.0984)
**5**	ASN90 (−0.9996) SER12 (−0.3889) GLY9 (−0.145) THR190 (1.0804)			PHE192 (−1.0039) ILE14 (−0.9349) CYS185 (−0.1611)	TYR155 (−0.5008) LYS159 (−0.2892) SER142 (−0.2522)
**6**	VAL188 (−1.233) GLY186 (−0.7452) TYR155 (−0.6104) SER142 (−0.2699) THR190 (0.5399)			PHE192 (−1.5739) ILE14 (−1.0434)	ASN90 (−0.9337) PHE226 (−0.238) THR140 (−0.2162) PRO187 (1.0923)
**8**	VAL188 (−1.1105) ASN90 (−0.4933) THR190 (0.7801)	TYR155 (−0.4568)		PHE192 (−1.5267) ILE14 (−1.0426)	THR140 (−0.3635) LYS159 (−0.2711) SER142 (−0.0517)
**9**	GLY94 (−0.9894) GLY92 (−0.811) SER11 (−0.6928) SER12 (9.0552)	LYS195 (−1.1394) TYR155 (−1.0113)	PHE192 (5.8101)	LEU93 (−1.8663) ILE14 (−1.7045) VAL196 (−1.0286) ALA106 (−0.3699) LEU95 (1.3807)	ASN90 (−2.3374) ALA91 (−1.5627) ARG37 (−0.5212) SER142 (−0.3955) CYS185 (−0.3436)
**14**	ASN90 (−0.8782) GLY92 (−0.822) SER12 (−0.5469)	ARG37 (−1.3972) SER11 (−0.4518) LYS159 (−0.3494)		PHE192 (−1.2886) ALA91 (−0.6477) VAL113 (−0.4438) VAL66 (−0.273)	LEU93 (−0.5816) THR140 (−0.3649) TYR155 (−0.3056)
**15**	GLY92 (−0.5283) GLY9 (−0.4326)	ARG37 (−1.8678) SER12 (−0.5819) THR190 (−0.2891)	PHE192 (−1.547)	VAL113 (−0.8616) ALA91 (−0.383) VAL66 (−0.2579)	LEU93 (−0.6114) ARG67 (−0.4835) ALA191 (−0.1625) ASP65 (−0.0007)
